# Effects of Leg-Length Discrepancy Compensation and Wedge Foot-Orthoses on Tensor Fasciae Latae EMG in Runners

**DOI:** 10.3390/sports13110412

**Published:** 2025-11-17

**Authors:** Ruben Sanchez-Gomez, Boon Peng Chang, Vitali Lipik, Paola Sanz-Wozniak, Dan Iulian Alexe, Jimena Garrido Cebrecos, Marta Martín Vega, Alvaro Gomez Carrion

**Affiliations:** 1 Nursing Department, Faculty of Nursing, Physiotherapy and Podiatry, Universidad Complutense de Madrid, 28040 Madrid, Spain; rusanc02@ucm.es (R.S.-G.); martma51@ucm.es (M.M.V.); alvaroalcore@hotmail.com (A.G.C.); 2 Instituto de Investigación Sanitaria Hospital Clínico San Carlos (IdISSC), 28040 Madrid, Spain; 3 School of Materials Science and Engineering, Nanyang Technological University, 50 Nanyang Avenue, Singapore 639798, Singapore; vitali@ntu.edu.sg; 4 Clínica Pododinámica, 28005 Madrid, Spain; paolsanz@ucm.es (P.S.-W.); jimenagarrido98@gmail.com (J.G.C.); 5 Instituto de Ciencia y Tecnología de Polímeros (ICTP), Centro Superior de Investigaciones Científicas (CSIC), 28006 Madrid, Spain; 6 Department of Physical and Occupational Therapy, Faculty of Movement, Sports and Health, Sciences, “Vasile Alecsandri” University of Bacău, 600115 Bacău, Romania; alexedaniulian@ub.ro

**Keywords:** leg length discrepancy, foot, orthoses, EMG

## Abstract

Aims: Structural lower limb-length discrepancies (LLLD) have been classically associated with the etiology of low back pain. However, their biomechanical effects on lower-limb muscle activity during running remain unclear. This pilot crossover study aimed to evaluate the influence of orthotic interventions—designed to compensate for LLLD and modify foot biomechanics—on the electromyographic (EMG) activity of the contralateral tensor fasciae latae (TFL) in healthy runners. Methods: A total of 41 recreational male and female runners (mean age 32.27 ± 6.09) with structural LLLD were recruited and classified as neutral (Ng), supinated (SPg), or pronated (PRg) based on their foot posture. Surface EMG activity of the TFL in the longer leg was recorded with specific surface electrodes while participants ran on a treadmill at a constant speed of 9 km/h for 3 min. Each subject randomly wore standard orthoses with 5 mm pronating (PRO), supinating (SUP) wedges or orthoses with a heel lift (TAL) to compensate for the shorter leg, alongside the baseline condition (SIN). Results: Perfect reliability (close to 1) was obtained for all measurements. A statistically significant reduction in TFL EMG activity was recorded in the Ng group: SIN 105.64 ± 50.6%MVC vs. PRO 100.16 ± 48.61%MVC (*p* < 0.05), and SIN vs. TAL 93.49 ± 15.88%MVC (*p* < 0.001). A significant reduction was also observed in the PRg group: SIN 91.82 ± 40.75%MVC vs. TAL 80.08 ± 31.75%MVC (*p* < 0.05). Conclusion: Orthotic compensation for LLLD and foot pronation modifications produced measurable changes in TFL EMG activity during running. These findings provide mechanistic insight into the interaction between limb-length asymmetry, foot biomechanics, and proximal muscle activation in runners, and may inform future studies on overuse injuries such as iliotibial band syndrome.

## 1. Introduction

Iliotibial band (ITB) syndrome is an overuse injury that causes lateral knee pain and affects a growing number of recreational runners each year [1]. It is the most common cause of lateral knee pain in this population [2]. ITB syndrome is a multifactorial condition in which both external factors (e.g., training intensity and volume) and internal variables (e.g., biomechanics) are involved. Traditionally, it has been explained as a friction phenomenon caused by the posterior displacement of the ITB over the lateral femoral condyle during knee flexion and extension [3,4]. However, a study [5] suggested that when the knee flexes beyond 30°, the ITB compresses medially over the fat pad, blood vessels, nerves, and Pacinian corpuscles located at its insertion on the lateral femoral epicondyle. This compressive mechanism is now considered the true cause of pain, rather than friction or gliding over the femur [5].

Understanding the pathology of ITB syndrome requires a review of the functional anatomy of the structure. The ITB connects to the hip via the tensor fasciae latae (TFL), which originates from the anterior iliac crest and partially integrates fibers from the anterior portion of the gluteus medius (GMed) [6,7]. Therefore, the ITB–TFL–GMed complex plays a key role in maintaining pelvic stability by acting as a lateral support during single-leg phases of gait—such as the swing phase in walking or running—and is crucial for controlled deceleration [8,9]. Additionally, due to its secondary insertions on the lateral patella and femur, along with its primary attachment on the tibia, the ITB contributes significantly to knee joint stabilization during running [10]. Structural imbalances, such as lower limb-length discrepancies (LLLD), may alter force distribution and muscular equilibrium between the left and right hip joints, potentially leading to knee disorders [11,12,13]. While the association between LLLD and low back pain is well established [14], its specific impact on the electromyographic activity of hip musculature during gait remains unclear. Given the lateral position of the thigh musculature and the elevation caused by LLLD on one side, we hypothesize that such imbalance could be a contributing factor to the development of ITB syndrome.

Furthermore, since lateral hip muscles are responsible for both hip abduction and adduction, several studies have suggested a relationship between increased hip adduction moments, weakness in the abductors (such as the GMed and TFL), and ITB syndrome in runners [1,14,15,16]. Foot biomechanics also play a critical role. Both excessive foot supination [17,18] and excessive pronation [19,20] during gait affect the alignment and function of the entire lower limb. In fact, overpronation has traditionally been linked to ITB syndrome through its association with excessive internal rotation and adduction of the lower extremity [8,21,22,23]. If structural varus alignment is considered a predisposing factor [11,24], then excessive supination may represent an under-recognized contributor to ITB pathology in runners. D’Amico et al. speculated that LLLD could lead to global biomechanical alterations throughout the kinetic chain [25]. Nevertheless, there is still limited evidence concerning the compensatory response of the TFL to LLLD during running, despite its known synergistic function with the ITB [26,27].

Surface electromyography (EMG) has proven to be a valid and reliable tool for assessing muscular activity in the hip, including the gluteus medius and maximus, under various conditions [28,29,30]. Neither the biomechanical effects of orthotic compensation for overpronation/supination nor the influence of LLLD on TFL EMG activity during running have been studied to date. Therefore, the purpose of this study was to analyze changes in TFL EMG activity in neutral (Ng), supinated (SPg), and pronated (PRg) foot types when using orthoses that induce pronation (PRO), supination (SUP), or a heel lift (TAL) to compensate for the shorter leg, compared to a baseline condition (SIN). We hypothesized that correcting limb-length discrepancies and counteracting the biomechanical effects of excessive supination would reduce EMG activation in the TFL and, consequently, relieve strain on the ITB—offering a novel approach to managing ITB syndrome.

## 2. Material and Methods

### 2.1. Sample Size and Study Design

This research was designed as a pilot crossover study, corresponding to Level II evidence. The study received approval from the designated Ethics Committee (Ref. C.I. 22/740-E). All procedures involving human participants were conducted in accordance with the ethical principles outlined in the Declaration of Helsinki (1964). Prior to participation, written informed consent was obtained from all individuals involved.

In a comparable study [31] on hip muscle EMG activity, recorded values were 97.61 ± 50.43%MVC for concentric and 42.99 ± 25.89%MVC for eccentric contractions (mean ± SD; *p* < 0.001). Using these data, a statistical power of 80%, a 95% confidence interval (CI), β = 0.20, and α = 0.05 were applied to estimate a required sample size of 10 participants per group to detect a minimum difference of 33–42%MVC in mean EMG activity using ANOVA for independent samples. To account for possible dropouts, the final target sample was set at 11 participants per group, and the total number of participants was 41.

This analytical crossover study followed the STROBE guidelines for reporting observational studies [32]. Randomized consecutive sampling was used, and the study was conducted between April 2023 and February 2024.

### 2.2. Participants

The participants were selected based on a set of carefully defined criteria to ensure a homogeneous sample and minimize potential confounding factors. The general inclusion criteria included: (1) healthy men and women aged between 18 and 38 years; (2) recreational rearfoot strike pattern runners with a minimum of 20 km of weekly training (2–3 days per week) and at least 1 year of running experience [31]; (3) experience running on a treadmill; (4) the presence of structured (anatomical) congenital or acquired lower limb-length discrepancy (LLLD) [33], assessed through direct measurement from the anterior superior iliac spine to the medial tibial malleolus [3], within a range of 7 mm to 15 mm [34]; (5) body mass index (BMI = weight [kg]/height [m^2^]) within an acceptable range; and (6) foot size between 38 and 42 to avoid potential bias in data collection or result interpretation [35]. In addition, specific inclusion criteria were applied according to the differentiated groups [36]: (1) Ng participants were required to have Foot Posture Index (FPI) scores between 0 and 5; (2) PRg participants had FPI scores between +6 and +9; and (3) SPg participants had FPI scores between −1 and −4. Participants were excluded if they presented: (1) any pain or lower limb/foot disorder, or any injury at the time of testing or within the previous year; (2) any mobility restriction in the lower limb beyond valid values [21,37,38,39,40,41,42,43,44]; (3) if they were under the effects of any medication at the time of the test; or (4) if their FPI scores fell outside the indicated range for their respective group.

### 2.3. Instruments and Assessments

A NeuroTrac Simplex Plus^®^ (Verity Medical Ltd., Braishfield, UK) electromyographic surface EMG device, validated in previous studies [38,39], was used to assess the EMG activity of the TFL during the treadmill running activities while wearing the different orthoses, randomly assigned. The device had a recording range of 0.2%MVC to 2000% MVC, with an accuracy of 4% of the reading from %MVC ± 0.3%MVC up to 200 Hz. It featured a bandpass filter from 18 Hz ± 4 Hz to 370 Hz ± 10 Hz for readings below 235%MVC and a sensitivity of 0.1%MVC root mean square (RMS). Artifacts were managed by visual inspection and rejection. Its wireless connection range was 10 m via Bluetooth. Self-adhesive circular surface electrodes were placed on the TFL area of the longer leg. These electrodes measured 30 mm (mm) in diameter and were made of high-quality hydrogel and conductive carbon film. The signal was received by the module and automatically filtered through the NeuroTrac^®^ software. V3.0 The data was then securely transmitted to the computer via a unidirectional radiofrequency connection, where the software converted it into digital data displaying the activity patterns and measurement data for each electrode.

### 2.4. Materials

The orthotic devices used in this study were manufactured using a standard sheet of ethylene-vinyl acetate (EVA), a material widely employed in podiatric practice due to its semi-rigid properties and biomechanical neutrality. Each orthotic base was 1 mm thick, ensuring that the device did not interfere with the natural function of the foot or alter baseline gait parameters. These standard orthoses served as the foundation upon which the specific modifications intended to induce pronation, supination, or heel elevation were added.

To simulate foot supination, a 5 mm wedge made of rigid EVA was affixed to the medial aspect of the rearfoot region of the orthosis. This configuration encouraged inversion of the calcaneus and external rotation of the limb. Conversely, for the pronated condition, the same type of 5 mm EVA wedge was attached laterally, promoting eversion of the rearfoot and facilitating internal rotation. For the limb-length compensation condition, a 5 mm [38] heel lift was fabricated from the same rigid EVA material and placed under the entire rearfoot area of the orthosis corresponding to the shorter leg. This lift aimed to restore pelvic symmetry during the stance phase of gait.

To maintain experimental rigor and avoid any potential biomechanical imbalances during testing, each participant wore the same neutral flat insole on the contralateral foot, except in the TAL (heel lift) condition. In that case, the TAL orthosis was applied to the shorter limb, while the contralateral foot used the baseline flat insole (SIN) to ensure a controlled comparison.

All orthotic devices were manufactured by an external, specialized orthoses laboratory (Termofeet^®^ SL, Madrid, Spain), which remained blinded to all experimental procedures. The neutral sports shoes used in this study were a standard commercial model (‘New Feel PW^®^ 10 M’, medium gray color, reference number: 20182022, Decathlon SA, Villeneuve-d’Ascq, France), ensuring consistency across participants and testing conditions.

### 2.5. Procedure

All measurements and procedures were conducted by the same sports podiatry specialist (RSG), who was also one of the study’s authors. The initial step involved assessing the presence and magnitude of the LLLD in each participant. To identify anatomical inequalities, the Galeazzi test—also known as the Allis test or, in its extended form, the Weber-Barstow maneuver—was performed [33]. Once a discrepancy was confirmed, the clinician determined which limb was shorter by measuring the linear distance from the anterior superior iliac spine to the medial malleolus, following the method described by Beattie et al. [3].

#### 2.5.1. Electrodes Placement

After identifying the shorter limb, the placement of EMG electrodes was carried out. The target for EMG data collection was the TFL muscle on the longer limb, as this side typically bears more biomechanical demand due to pelvic imbalance. Electrode placement followed the internationally recognized SENIAM protocol (Surface ElectroMyoGraphy for the Non-Invasive Assessment of Muscles, available at http://www.seniam.org (12 April 2023). The active electrodes were aligned longitudinally with the orientation of the muscle fibers in the TFL belly. To accurately locate the muscle belly, each participant lay in lateral decubitus on their shorter side. They were instructed to perform an upward movement (hip abduction) with the longer leg, which was positioned on top. The clinician applied resistance to this movement to cause maximal contraction of the TFL, thereby allowing for clear palpation and anatomical marking of the muscle belly. Two circular self-adhesive surface electrodes were then placed on the TFL, while the neutral (reference) electrode was positioned over the vastus lateralis, as per the manufacturer’s guidelines. Before initiating the treadmill tests, each participant was asked to perform a maximal voluntary isometric contraction (%MVCC) of the hip abductors by pushing against the clinician’s manual resistance for a period of 5 s. This calibration step was necessary to normalize the EMG signals during data processing, enabling accurate comparisons between conditions and among participants.

#### 2.5.2. Runing Test

The running trials were performed on a motorized treadmill (Domyos^®^ T520). Prior to data collection, each participant completed a 3 min warm-up at 5.7 km/h to become accustomed to the treadmill surface and to reduce any potential biomechanical changes caused by unfamiliar footwear or materials [31,35,40]. This familiarization step ensured that participants were prepared for the subsequent experimental procedures. Following the acclimation phase, participants completed four test conditions—SIN, PRO, SUP, and TAL— in a randomized order and blinded, without knowing which orthoses were inside. In each condition, they ran for 3 min at a constant speed of 9 km/h, which was chosen based on prior EMG studies [38] to ensure standardized, submaximal running effort [10]. To minimize the influence of fatigue, a 5 min passive recovery period was provided between each trial [41]. Each participant completed a total of 12 tests (three trials per condition). Throughout all conditions, the PRO and SUP orthosis were placed on the longer limb and the non-test foot always wore a neutral flat insole to ensure biomechanical symmetry, except when the TAL orthosis was used, in which case the heel lift was placed on the shorter limb, and the contralateral foot was fitted with the SIN condition. All participants reported a low or negligible sensation of fatigue during and after the trials, ensuring that all subjects were working at a “light” intensity level.

This controlled protocol allowed for consistent EMG data collection under reproducible biomechanical conditions, accounting for limb dominance, foot posture, and orthotic compensation strategy.

### 2.6. Statistical Analysis

The anthropometric and demographic data of the participants were described using means and standard deviations (±SD). To ensure the reliability of the EMG measurements obtained during the treadmill running tests, the intraclass correlation coefficient (ICC) and the standard error of measurement (SEM) were calculated for each of the three participant groups (Ng, SPg, PRg) under the four experimental conditions (SIN, SUP, PRO, TAL). These values were used to evaluate the consistency of repeated measures conducted within the same day.

The ICC values were interpreted according to the classification proposed by Koo et al. [42,43], in which values below 0.50 indicate poor reliability; values between 0.50 and 0.75 indicate moderate reliability; values between 0.75 and 0.90 indicate good reliability; and values greater than 0.90 indicate excellent reliability. For the purposes of scientific rigor in this study, ICC values equal to or greater than 0.90 were considered optimal, indicating high reliability of the EMG data.

The SEM was used to determine the minimum detectable change (MDC) for each condition and group. Additionally, the Shapiro–Wilk test was employed to evaluate the normality of the sample distributions; a *p*-value greater than 0.05 was considered indicative of a normal distribution. To assess potential differences in TFL EMG activity across the orthotic conditions within each foot-type group, a Linear Mixed-Effects Model (LMEM) was employed, extending the analysis beyond a traditional repeated-measures ANOVA. The LMEM included condition, foot group, and their two-way interaction as fixed effects, with subject incorporated as a random intercept to account for the non-independence of repeated measures and the high inter-subject variability inherent in EMG data. When significant main or interaction effects were detected, pairwise comparisons were conducted on the Estimated Marginal Means using Tukey’s HSD (or similar adjustments such as Bonferroni) post hoc tests to precisely locate differences and control the family-wise error. LMEM estimates were calculated as (EMG SIN—EMG Condition), with a positive estimate (+) indicating a reduction in TFL activity (orthosis beneficial) and a negative estimate (−) indicating an increase in TFL activity (orthosis detrimental). *p*-values were derived from the LMEM post hoc contrasts. Post hoc effect sizes (Cohen’s drm) were calculated for paired contrasts to quantify the magnitude of observed differences.

**Figure 1:**
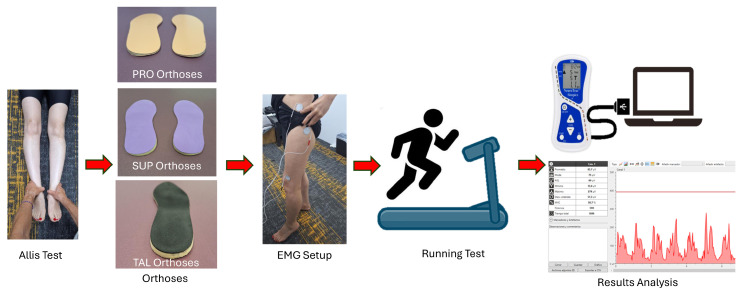
Schematic diagram illustrating the detailed procedure for the assessment and testing process.

The methodological procedure and testing sequence are presented in the schematic diagram shown in Figure 1.

## 3. Results

A total of 58 volunteers (32 women and 26 men) were initially recruited for the study. After applying the inclusion and exclusion criteria, 12 individuals were excluded due to noncompliance with the eligibility criteria, and 5 participants were lost to follow-up. As a result, a final sample of 41 participants was included in the analysis and all other data were expunged; the participants were distributed as follows: 14 in the Ng, 11 in the SPg, and 16 in the pronated group PRg.

The demographic characteristics of the participants are summarized in Table 1. The mean age of the total sample was 32.27 ± 6.09 years. The average height was 170.37 ± 8.15 cm, the mean weight was 64.04 ± 9.71 kg, and the average BMI was 21.81 ± 1.36 kg/m^2^. Foot size, based on European size, ranged from size 38 to 42, with a mean of 40.66 ± 1.9.

The Shapiro–Wilk test confirmed that the distribution of EMG data was normal in all groups and conditions (*p* > 0.05). The reliability analysis of the EMG measurements showed excellent consistency across repeated trials. ICC values for all conditions and participant groups ranged from 0.998 to 1.000, indicating near-perfect agreement (Table 2). The lowest ICC value was observed in the TAL condition of the Ng group (0.998), while the highest values (1.000) were recorded in multiple conditions across groups. The MDC varied depending on the group and orthotic condition. The highest MDC value was recorded in the Ng group with TAL orthoses (5.79%MVC), whereas the lowest was in the SPg group with SUP orthoses (1.35%MVC), as shown in Table 2. As previously described, a total of 41 participants were included in the final analysis: 14 in the Ng, 11 in the SPg, and 16 in the PRg. Their anthropometric characteristics are detailed in Table 1. The normality of the EMG data distributions was confirmed for all groups using the Shapiro–Wilk test (*p* > 0.05).

To assess the consistency of the EMG signal recordings, the ICC and SEM were calculated for each group and experimental condition; for all analyzed data, the obtained ICCs showed excellent reliability; these data are presented in detail in Table 2.

Comparative analysis of TFL EMG activity across the four orthotic conditions (SIN, SUP, PRO, TAL) using the LMEM (Table 3) showed a general trend toward reduced EMG activity (%MVC) with the use of insoles compared to the SIN condition across all foot groups. Although most contrasts were not statistically significant, the direction of change consistently indicated lower muscle activation with orthotic use, except for the SIN vs. SUP contrasts in the Ng (6.35 MVC%) and PRg (1.13%MVC), which showed slight increases.

Statistically significant differences were found in the SPg for SIN vs. PRO (*p* < 0.05) and SIN vs. TAL (*p* < 0.001), indicating a significant reduction in EMG activity when using pronated and heel lift orthotics. Additionally, when comparing the EMG activity in the SIN condition between the three foot-type groups, the SPg exhibited significantly higher baseline EMG activity (140.02 ± 40.84%MVC) compared to the PRg (91.82 ± 40.75%MVC). This suggests that runners with supinated feet may experience greater muscular demand in the TFL during running, even without any orthotic intervention.

The extensive post hoc analysis (Table 4) confirms that the significant differences reported in TFL EMG activity are not only statistically significant but also represent a large to very large magnitude of effect (drm ≥ 1.14) across the most critical orthotic interventions in all three foot-type groups. Specifically, the TAL orthosis demonstrated a very large effect on TFL deactivation in both the Ng (drm = 1.89) and PRg (drm = 2.15) groups, while the SUP orthosis showed the largest positive effect in the SPg group (drm = 2.26).

The within-subject analysis of TFL EMG activity demonstrated a consistent pattern of muscular deactivation following specific orthotic interventions (Table 5). In the Ng, the greatest average reduction in TFL activity relative to the baseline condition SIN was observed with the TAL orthosis (10.58% MVC), supporting the effectiveness of compensating the LLLD in this population. Similarly, the PRg showed its maximum TFL deactivation with the TAL (−6.73%MVC), while the SUP resulted in a deleterious increase (+6.66%MVC).

**Figure 2:**
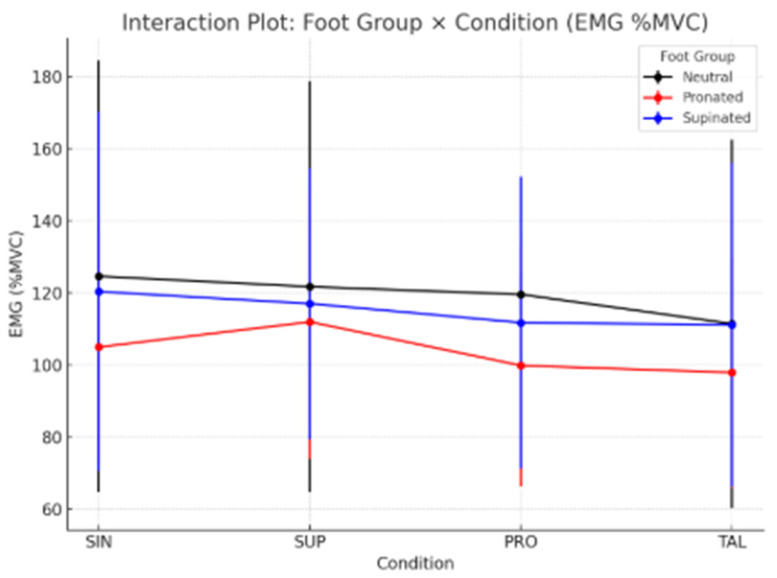
Schematic Interaction Plot illustrates the detailed procedure for the assessment and testing process.

The interaction plot (Figure 2) shows distinct EMG response patterns across foot groups and conditions. Ng (black line) and SPg (blue line) exhibited a progressive reduction in EMG activity from SIN to TAL, while the PRg (red line) showed a slight increase under the SUP condition followed by a decrease, suggesting different adaptation mechanisms among foot types.

## 4. Discussion

The main objective of the present study was to evaluate the EMG activity of the TFL when using different types of foot orthoses during running, in order to analyze its specific responses in relation to foot type and its compensatory role in participants with LLLD. To date, no studies have reported these specific characteristics in a running context, nor have they explored their relationship with ITB syndrome, LLLD, or foot type. Only a recent systematic review [44] has recommended the use of orthoses and biomechanical interventions—such as podiatric assessment and correction of leg-length discrepancies—as adjuvant therapies, although without providing detailed guidance.

A recent study by Benito et al. [28] simulated LLLD in healthy participants by applying different heel lifts while subjects walked. The EMG activity of Gmed was assessed on the same leg where the lifts were applied, and the results showed a significant decrease in EMG activity as the lift height increased. If we assume a compensatory increase in EMG activity on the contralateral, artificially lengthened limb [45], our findings may be consistent with theirs, as the EMG activity of the TFL in the longer leg in our study decreased significantly after compensation with TAL on the shorter limb in all groups. These results are also in agreement with another study [46] that reported increased EMG activity of the quadriceps muscle in the artificially lengthened leg. Based on this reasoning and our findings, we suggest that compensating for the shorter limb may help reduce muscular fatigue in the contralateral hip musculature, which plays a key role in maintaining postural balance [47]. Further research is needed to explore this issue in greater depth, with the aim of preventing a major cause of morbidity in older populations: falls related to muscular weakness and instability.

Focusing on runners, it has been demonstrated that the most effective way to reduce TFL neuromuscular demand in all groups (SPg, PRg, and Ng) was by balancing LLLD with a TAL on the shorter limb, which contributed to preserving the integrity of the ITB—more effectively, in fact, than compensating for foot type alone. Previous studies [14,48] have suggested that maintaining proper lateral hip balance in athletic populations may help prevent a wide range of lower limb injuries, such as ITB syndrome, since one of the hip’s primary functions is to absorb impact forces during the early stance phase of running. Moreover, compensating for LLLD has been hypothesized as an effective strategy to preserve running economy [49,50]. Given their short lever arms but considerable strength, the hip muscles are particularly prone to overuse injuries [18]. A recent review [51] has also emphasized that all scoliotic discrepancies should be compensated for when the deformity of the femoral head occurs on the same side as the pelvic and sacral tilt.

On the other hand, classical literature [22,52,53,54] has shown that rearfoot overpronation was considered a mechanism responsible for insufficient impact attenuation against ground reaction forces and was associated with poor control of excessive and rapid internal knee rotation during running. Under the traditional theory that attributed ITB syndrome to friction and translation mechanisms, this explanation was plausible. However, with the emergence of new concepts such as fat pad enrichment, increased vascularization, and nerve compression [6], this theory no longer offers a specific or complete explanation. Instead, the bowstring effect associated with dynamic and/or structural knee varus is now considered a more accurate pathomechanical explanation. To date, no studies have examined the EMG activity of the TFL in runners with LLLD in relation to foot type. We hypothesized that reducing the structural varus of the lower limb through foot pronation—i.e., promoting internal rotation of the leg with PRO—would decrease TFL EMG activity and, consequently, reduce the strain along the course of the ITB. The SPg demonstrated a significant reduction in neuromuscular demand when using PRO, and a highly significant reduction when using TAL, both compared to the SIN condition. This suggests that a more varus limb structure, or dynamic supinated movement patterns involving external rotation, impose greater strain on the ITB, thus supporting our hypothesis. Although there are no specific studies with which to directly compare our data, our findings are consistent with conclusions indicating that dynamic genu varus is one of the etiological factors contributing to ITB syndrome [11,24]. They also align with previous research [18] suggesting that a supinated foot, due to its poor shock-absorbing capacity, may contribute to knee injuries in runners. Furthermore, several biomechanical reviews have highlighted the lack of evidence supporting overpronation as a causal factor in ITB syndrome [55], as well as similar mechanisms related to pronation—such as hip internal rotation [23,44], knee adduction [10,22], or hip adduction [2,22]—as injury catalysts in the development of this condition. These discrepancies were likely due to differences in assessment methods, study population selection criteria, and the ability to accurately diagnose ITB syndrome and other knee pathologies [10].

Our findings highlight that foot type and lower limb-length discrepancies can influence pelvic alignment and can increase strain on the ITB. Compensating these biomechanical imbalances through the use of foot orthoses may reduce mechanical stress on the ITB. The consistency and magnitude of these results reinforce their strong clinical interpretability, indicating that the orthopedic interventions produced meaningful reductions in the neuromuscular demand of ITB. Overall, these findings support a more holistic approach to the management of ITB syndrome—one that extends beyond knee-focused treatments to include comprehensive assessments of the lower limb and foot in clinical practice.

Practical Contributions

- The findings of this study advance current knowledge on the treatment of iliotibial band (ITB) syndrome in runners by moving beyond the traditional, knee-centered approach.
- Foot type, in combination with lower limb-length discrepancies, influenced pelvic alignment and, consequently, increased strain on the iliotibial band. By correcting these biomechanical imbalances through the use of foot orthoses, it would be possible to reduce mechanical stress on the ITB and promote faster recovery from the condition.

### Limitations and Futures Lines of Investigation

The aim of this study was neither to evaluate specific degrees of LLLD nor to provide individualized treatments; accordingly, the precise magnitude of LLLD in the participants was not considered. Instead, subjects were simply classified as having LLLD, forming a heterogeneous group with discrepancies ranging from 7 to 15 mm. All were compensated with a standard 5 mm TAL orthotic, regardless of the exact extent of their limb length discrepancy. The authors acknowledge that compensating the LLD with a personalized TAL for each subject could be more accurate. However, it is argued that even if the compensation was not fully individualized, improvements in TFL EMG would still be expected using the standard 5 mm TAL. This approach may have contributed to the variability in EMG activity responses and to the heterogeneity of the recorded values among participants. Moreover, sensitivity or subgroup analyses based on true LLD were not performed, as the study aimed to assess general TFL EMG responses to standardized orthotic interventions. Future studies should address these questions to increase the robustness of the findings.

Based on the current literature [20], differences in gender, age, or dominant leg between groups were not considered in the present study. Further research could examine these covariates to explore additional factors affecting TFL function or pathology.

Future studies should investigate the combined effects of applying TAL on the shorter limb and PRO on the longer one during running gait, in order to assess their potential benefits in reducing EMG neuromuscular demand in the TFL. In addition, future studies should include step recognition to analyze muscle activation by gait phase, allowing a more detailed interpretation of EMG patterns.

Given the high minimal detectable difference (33–42%MVC), the study’s statistical sensitivity is limited; therefore, the results should be interpreted with caution and considered exploratory. In addition, given the high variability in strength among subjects, the standard deviations of the measurements obtained were markedly elevated.

Including only participants with shoe sizes EU 38–42 may introduce a selection bias. However, this restriction was necessary to homogenize the sample, as foot size strongly influences running biomechanics.

## 5. Conclusions

ITB syndrome is a common cause of activity limitation in runners due to knee pain. Its classical association with foot type has been little studied previously, and the conclusions have been inconclusive. Likewise, its relationship with hip imbalance has been described, although not specifically in relation to LLLD. The present study aimed to evaluate the effectiveness of TAL compensation and foot-related orthoses in reducing EMG neuromuscular demand of the TFL, a muscle closely associated with ITB syndrome in runners. The results suggested that compensating the shorter limb with TAL is the most effective strategy for reducing neuromuscular demand in the contralateral TFL, thereby offering a promising approach to relieving tension on the ITB and addressing its associated pathology. Additionally, promoting rearfoot pronation in the longer limb during running appears to be another viable strategy for reducing EMG activity of the TF as it may help counteract the bowstring effect associated with functional or structural genu varus of the knee.

## Figures and Tables

**Table 1:** Subject Demographics.

	Total Population *n* = 41	Neutral Group (Ng) *n* = 14	Supinated Group (SPg) *n* = 11	Pronated Group (PRg) *n* = 16
Variable	mean ± SD (95% CI) %	mean ± SD (95% CI) %	mean ± SD (95% CI) %	mean ± SD (95% CI) %
Age (years)	32.27± 6.09 (30.40–34.14)	32.71 ± 7.66 (28.68–36.74)	36 ± 3.63 (33.56–38.44)	28.12 ± 6.98 (24.40–31.84)
Height (cm)	170.37 ± 8.15 (167.88–172.86)	169.78 ± 9.32 (164.9–174.66)	170.09 ± 8.33 (165.15–175.0.3)	171.25 ± 6.8 (167.92–174.58)
Weight (kg)	64.04 ± 9.71 (61.14–66.94)	63.14 ± 7.87 (59–77.28)	65 ± 10.58 (58.75–71.25)	64 ± 10.7 (58.75–69.25)
Foot Size (Es)	40.66 ± 1.9 (40.05–41.27)	40.2 ± 2.1 (38.81–41.23)	39.3 ± 2.4 (41.69–43.31)	42.5 ± 1.2 (38.02–40.58)
BMI (kg/m^2^)	21.81 ± 1.36 (21.39–22.23)	21.9 ± 0.4 (21.69–22.11)	21.08 ± 2.3 (19.72–22.14)	22.46 ± 1.4 (21.77–23.15)

Abbreviations: SD = standard deviation; CI = confidence interval; FPI = foot posture index; BMI = body mass index; Es = number according to European mode size; cm = centimeters; Kg = kilograms; m^2^ = square meters.

**Table 2:** Reliability ICC and SEM of the Tensor Fascia Latae’s EMG values of variables of three groups in SIN, SUP, PRO and TAL interventions.

	Neutral Group (Ng) *n* = 14			Supinated Group (SPg) *n* = 11			Pronated Group (PRg) *n* = 16		
Intervention	mean (%MVC) ± SD (95% CI)	ICC 95% IC (Li-Ls)	MDC	mean (%MVC) ± SD (95% CI)	ICC 95% IC (Li-Ls)	MDC	mean (%MVC) ± SD (95% CI)	ICC 95% IC (Li-Ls)	MDC
SIN	105.64 ± 50.6	1 (0.999–1)	2.318	140.02 ± 40.84	1 (0.999–1)	2.25	91.82 ± 40.75	0.999 (0.997–0.999)	4.36
SUP	111.99 ± 50.9	1 (1–1)	1.8	139.52 ± 41.74	1 (1–1)	1.35	92.95 ± 46.83	1 (1–1)	1.84
PRO	100.16 ± 48.61	1 (0.999–1)	2.85	119.51 ± 35.89	0.999 (0.998–1)	2.42	86.73 ± 36.12	1 (0.999–1)	2.12
TAL	93.49 ± 15.88	0.998 (0.995–0.99)	5.79	105.81 ± 43.43	1 (0.999–1)	1.81	80.08 ± 31.75	1 (0.999–1)	1.72

Abbreviations: SD = standard deviation; CI = confidence interval; Li = lower limit; Ls = upper limit; MDC = minimum detectable change; ICC = intraclass correlation coefficient; %MVC = % maximal voluntary contraction; SIN = basal condition sport shoes; SUP = supinated orthoses; PRO = pronated orthoses; TAL = heel lift orthoses. Table 3 presents the comparative EMG data for the TFL during running activity across four different conditions for each group, along with the corresponding *p*-values.

**Table 3:** EMG signal amplitudes of Tensor Fascia Latae mean muscle activity for each group between the different study interventions.

Foot Group (*n*)	Contrast	Mean ±SD (%MVC) (95% CI)	LMEM Estimate (Mean Difference %MVC)	Standard Error (SE)	*p*-Value
Neutral (Ng) (*n* = 14)	SIN	105.64 ± 50.60	Base	Base	Base
	SIN vs. SUP	111.99 ± 50.90	−6.356	6.427	0.329
	SIN vs. PRO	100.16 ± 48.61	5.474	6.427	0.396
	SIN vs. TAL	93.49 ± 15.88	12.149	6.427	0.066
Pronated (PRg) (*n* = 16)	SIN	91.82 ± 40.75	Base	Base	Base
	SIN vs. SUP	92.95 ± 46.83	−1.136	8.081	0.889
	SIN vs. PRO	86.73 ± 36.12	5.091	8.081	0.534
	SIN vs. TAL	80.08 ± 31.75	11.739	8.081	0.157
Supinated (SPg) (*n* = 11)	SIN	140.02 ± 40.84	Base	Base	Base
	SIN vs. SUP	139.52 ± 41.74	0.501	8.873	0.955
	SIN vs. PRO	119.51 ± 35.89	20.514	8.873	0.031 *
	SIN vs. TAL	105.81 ± 43.43	34.217	8.873	0.001 **

Abbreviations: LMEM = linear mixed effects; SD = standard deviation; %MVC = maximal voluntary contraction; SIN = basal condition sport shoes; SUP= supinated orthoses; PRO = pronated orthoses; TAL = heel lift orthoses; *p*-value = level of significance; *p* < 0.05 * (with a 95% confidence interval) was considered statistically significant; *p* < 0.001 ** (with a 95% confidence interval) was considered statistically significant.

**Table 4:** Post Hoc Effect Sizes (Cohen’s drm) for Paired Contrasts.

Foot Group	Contrast	Mean DIF ± SD EMG (%MVC) (SIN—Condition)	drm	Interpretation (Cohen’s drm)
Neutral (Ng) (*n* = 11)	SIN vs. SUP	2.91 ± 5.99	0.49	Medium
	SIN vs. PRO	5.04 ± 3.58	1.41	Large
	SIN vs. TAL	13.18 ± 6.97	1.89	Very Large
Pronated (PRg) (*n* = 9)	SIN vs. SUP	−6.99 ± 3.16	−2.21	Very Large (Detrimental Increase)
	SIN vs. PRO	5.12 ± 4.48	1.14	Large
	SIN vs. TAL	7.07 ± 3.29	2.15	Very Large
Supinated (SPg) (*n* = 11)	SIN vs. SUP	13.34 ± 5.91	2.26	Very Large
	SIN vs. PRO	8.62 ± 5.7	1.51	Large
	SIN vs. TAL	9.22 ± 6.96	1.32	Large

Abbreviations: Mean DIF ± SD = Standard Deviation of the Difference; %MVC = maximal voluntary contraction; SIN = basal condition sport shoes; SUP = supinated orthoses; PRO = pronated orthoses; TAL = heel lift orthoses; drm = Cohen’s d within-subject

**Table 5:** Mean TFL EMG Activity and Within-Subject Percentage Change by Foot Type Across Orthotic Conditions.

Foot Group	Condition	mean ± SD (95% CI) EMG (%MVC)	Average % Change vs. SIN	Clinical Relevance (Summary)
Neutral (Ng) *n* = 11	SIN	124.63 ± 59.88	Base 0%	Control without intervention.
	SUP	121.72 ± 57.06	−2.33%	Slight reduction.
	PRO	119.59 ± 5.73	−4.05%	Significant decrease.
	TAL	111.45 ± 51.04	−10.58%	Largest reduction (LLLD compensation).
Pronated (PRg) *n* = 9	SIN	104.97 ± 32.41	Base 0%	Control without intervention.
	SUP	111.96 ± 37.95	6.66%	Increase (SUP would be contraindicated).
	PRO	99.85 ± 33.47	−4.88%	Decrease (supports pronation).
	TAL	97.90 ± 31.95	−6.73%	Best reduction effect.
Supinated (SPg) *n* = 11	SIN	120.37 ± 49.82	Base 0%	Control without intervention.
	SUP	117.03 ± 37.56	−3.10%	Slight reduction.
	PRO	111.75 ± 40.54	−7.16%	Reduction.
	TAL	111.15 ± 44.92	−7.66%	Reduction.

Abbreviations: SD = standard deviation; CI = confidence interval; %MVC = maximal voluntary contraction; SIN = basal condition sport shoes; SUP = supinated orthoses; PRO = pronated orthoses; TAL = heel lift orthoses.

## Data Availability

The data supporting the findings of this study are available from the corresponding author upon reasonable request.
